# Supplementary figures and images for: The role of Nrf2 in thyroid maturation and hormone synthesis in vertebrate models
Source: Life Sci Alliance. 2026 May 7;9(7):e202603687. doi: 10.26508/lsa.202603687 (PMC13153729; doi:10.26508/lsa.202603687)

**Table S1:** Primers sequences used for RT-qPCR analysis

**
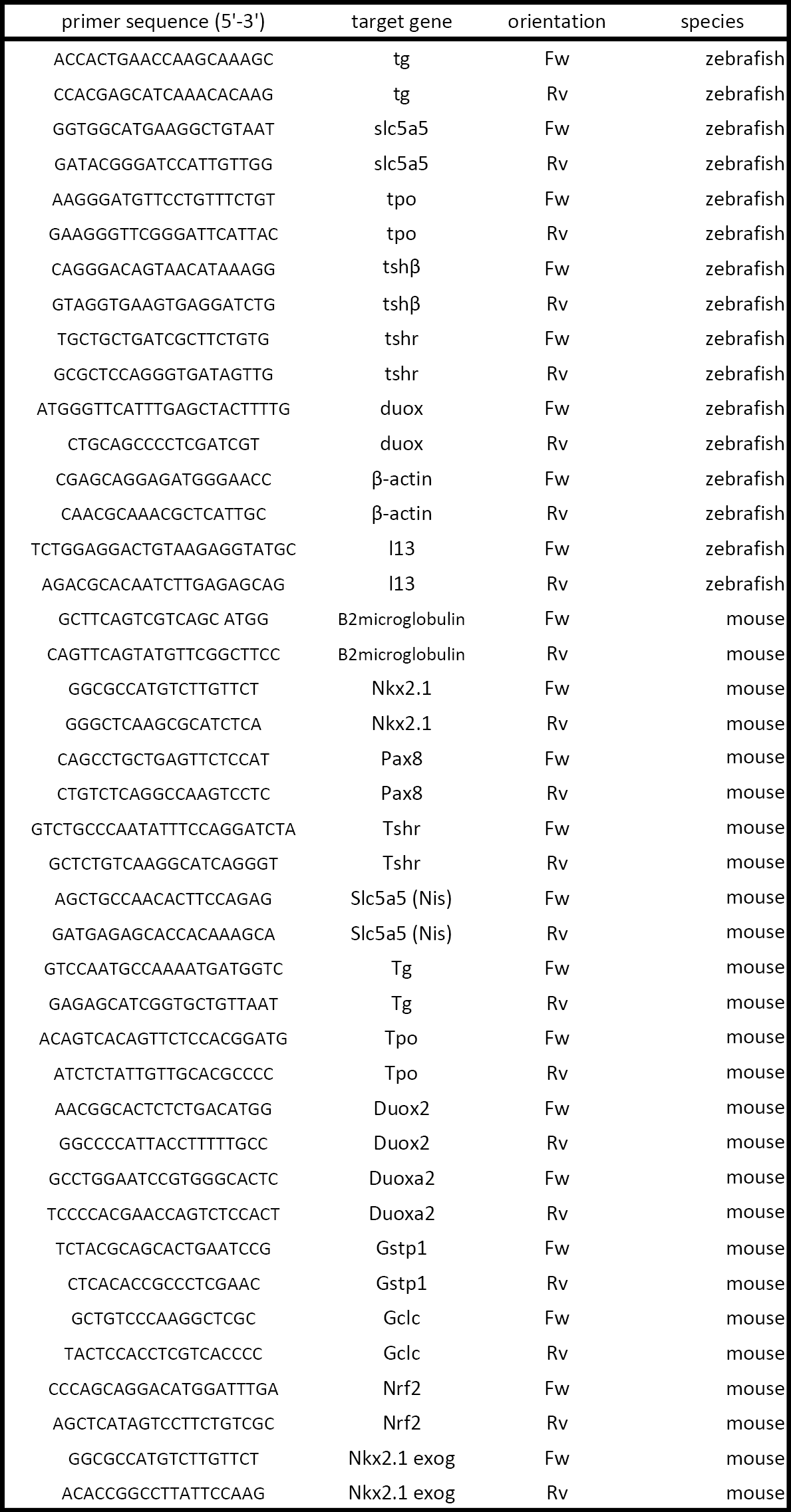
**

Supplement: Supplementary file 1 [file LSA-2026-03687_TableS1.docx]
